# European headache federation consensus on the definition of resistant and refractory migraine: Developed with the endorsement of the European Migraine & Headache Alliance (EMHA)

**DOI:** 10.1186/s10194-020-01130-5

**Published:** 2020-06-16

**Authors:** Simona Sacco, Mark Braschinsky, Anne Ducros, Christian Lampl, Patrick Little, Antoinette Maassen van den Brink, Patricia Pozo-Rosich, Uwe Reuter, Elena Ruiz de la Torre, Margarita Sanchez Del Rio, Alexandra J. Sinclair, Zaza Katsarava, Paolo Martelletti

**Affiliations:** 1 Neuroscience section – Department of Applied Clinical Sciences and Biotechnology, University of L’Aquila, Via Vetoio, 67100 L’Aquila, Italy; 2 Regional Referral Headache Center of the Abruzzo region, ASL Avezzano-Sulmona-L’Aquila, L’Aquila, Italy; 3 Headache Clinic, Department of Neurology, Tartu University Clinics, Tartu, Estonia; 4 Headache Unit, Neurology Department, Montpellier University Hospital and Montpellier University, Montpellier, France; 5 Department of Neurology, Headache Medical Centre Linz, Hospital Barmherzige Brüder, Centre of Integrative Medicine (ZiAM) Ordensklinikum Linz, Linz, Austria; 6 European Migraine & Headache Alliance (EMHA), Hendrik Ido Ambacht, The Netherlands; 7 Division of Pharmacology, Department of Internal Medicine, Erasmus MC University Medical Center Rotterdam, Rotterdam, The Netherlands; 8 Headache Unit, Neurology Department, Vall d’Hebron University Hospital, Barcelona, Spain; 9 Headache and Neurological Pain Research Group, Vall d’Hebron Research Institute, Department de Medicina, Universitat Autònoma de Barcelona, Barcelona, Spain; 10 Charité Universitätsmedizin Berlin, Department of Neurology, Charité Universitätsmedizin Berlin, Berlin, Germany; 11 Neurology department, Clinica Universidad de Navarra, Madrid, Spain; 12 Metabolic Neurology, Institute of Metabolism and Systems Research, College of Medical and Dental Sciences, University of Birmingham, Birmingham, UK; 13 Centre for Endocrinology, Diabetes and Metabolism, Birmingham Health Partners, Birmingham, UK; 14 Evangelical Hospital Unna, Unna, Germany; 15 Departmentof Neurology, University of Duisburg-Essen, Essen, Germany; 16 EVEX Medical Corporation, Tbilisi, Georgia; 17 IM Sechenov First Moscow State Medical University (Sechenov University), Moscow, Russian Federation; 18 Department of Clinical and Molecular Medicine, Sapienza University of Rome, Rome, Italy; 19 Regional Referral Headache Center of the Lazio region, Sant’Andrea Hospital, Rome, Italy

**Keywords:** Migraine, Chronic migraine, Resistant migraine, Refractory migraine, Intractable migraine

## Abstract

**Introduction:**

Despite advances in the management of headache disorders, some patients with migraine do not experience adequate pain relief with acute and preventive treatments. It is the aim of the present document to provide a definition of those migraines which are difficult-to-treat, to create awareness of existence of this group of patients, to help Healthcare Authorities in understanding the implications, and to create a basis to develop a better pathophysiological understanding and to support further therapeutic advances.

**Main body:**

Definitions were established with a consensus process using the Delphi method.

Patients with migraine with or without aura or with chronic migraine can be defined as having *resistant migraine* and *refractory migraine* according to previous preventative failures. *Resistant migraine* is defined by having failed at least 3 classes of migraine preventatives and suffer from at least 8 debilitating headache days per month for at least 3 consecutive months without improvement; definition can be based on review of medical charts. *Refractory migraine* is defined by having failed all of the available preventatives and suffer from at least 8 debilitating headache days per month for at least 6 consecutive months. Drug failure may include lack of efficacy or lack of tolerability. Debilitating headache is defined as headache causing serious impairment to conduct activities of daily living despite the use of pain-relief drugs with established efficacy at the recommended dose and taken early during the attack; failure of at least two different triptans is required.

**Conclusions:**

We hope, that the updated EHF definition will be able to solve the conflicts that have limited the use of definitions which have been put forward in the past. Only with a widely accepted definition, progresses in difficult-to-treat migraine can be achieved. This new definition has also the aim to increase the understanding of the impact of the migraine as a disease with all of its social, legal and healthcare implications. It is the hope of the EHF Expert Consensus Group that the proposed criteria will stimulate further clinical, scientific and social attention to patients who suffer from migraine which is difficult-to-treat.

## Introduction

Despite advances in the management of headache disorders, some patients with migraine do not experience adequate pain relief with acute and preventive treatments. This correlates with higher burden and disability, as well as despair of patients who suffer from it. The terms “refractory” and “intractable” headache have been used to describe this particular condition and various definitions have been suggested over time [1–6] (Table 1). However, wide acceptance of the proposed definitions was not reached so far. Moreover, the International Classification of Headache Disorders (ICHD) neither includes a definition for refractory or resistant migraine nor for other primary headaches [7].

**Table 1:**
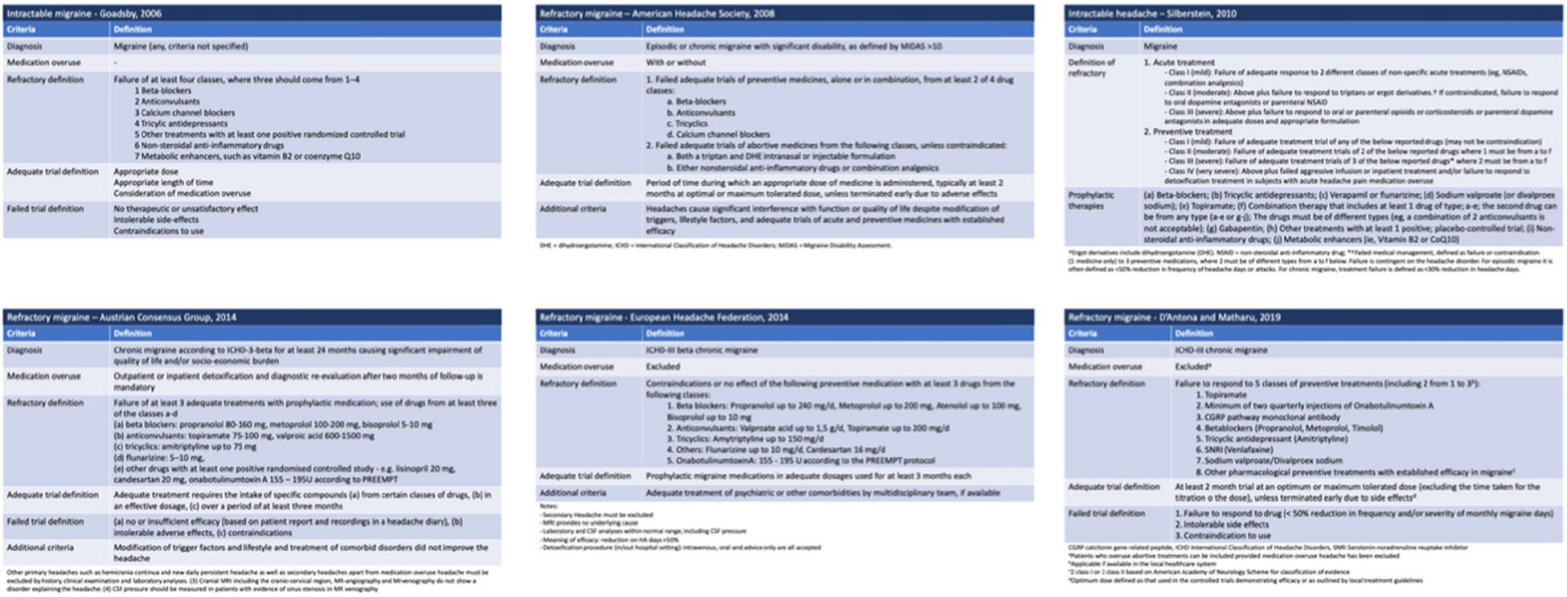
Available definitions for migraine which is difficult to treat

A previous consensus statement of the European Headache Federation (EHF) defined as refractory migraine those chronic migraine patients who do not show response to adequate dosages of at least 3 drugs from the following classes: beta-blockers, anticonvulsants, tricyclics, onabotulinumtoxinA and others (e.g., flunarizine, candesartan) for at least 3 months each, in absence of medication overuse [3]. The aim of the present Consensus paper is to critically revise the definition of those migraines which are difficult-to-treat, to create awareness of existence of this group of patients, to help Healthcare Authorities in understanding the implications, and to create a basis to develop a better pathophysiological understanding and to support further therapeutic advances.

## Methods

The Panel who developed this consensus statement consisted of the members of the Council of the EHF. Ten of the board members are physicians (with specialization in Neurology or Internal medicine) and one is a pharmacologist; all are experienced in headache. The Panel includes also two representatives (PL, ERDLT) of patients from the European Migraine and Headache Alliance (EMHA). Patients representatives were not involved in the consensus process to develop the definitions but had the opportunity to provide suggestions and comments.

This Consensus represents an update of a previous EHF consensus published in 2014 [3]. All the points of the previous EHF consensus definition were reconsidered by the EHF Expert Consensus Group and revised if necessary.

As a first step, an in-person meeting was held to agree on the need of a new Consensus Statement, on the composition of the EHF Expert Consensus Group and on the mission of this updated Consensus Statement. Thereafter, the process proceeded via e-mails.

The Delphi method [8] was used to reach consensus. According to this method, the EHF Expert Consensus Group members were assigned to open or multiple-choice questions in several rounds. Participants were instructed not to discuss the responses amongst themselves. Feedback after each round was sent only to the facilitator (SS). The facilitator collected all the answers for each round and issued an anonymized report with comments and agreement rates. Participants were then encouraged to revise their earlier answers in light of the replies of other members. Questions were repeated until a clear majority (> 70% of agreement) was reached [9]. Each Panel member (with the exclusion of EHMA representatives) had the right to vote. A step by step approach was used to build the definitions, using the results of the Delphi questions. Seven rounds were needed to reach a consensus among all the different aspects of the definition (Table 2).

**Table 2:**
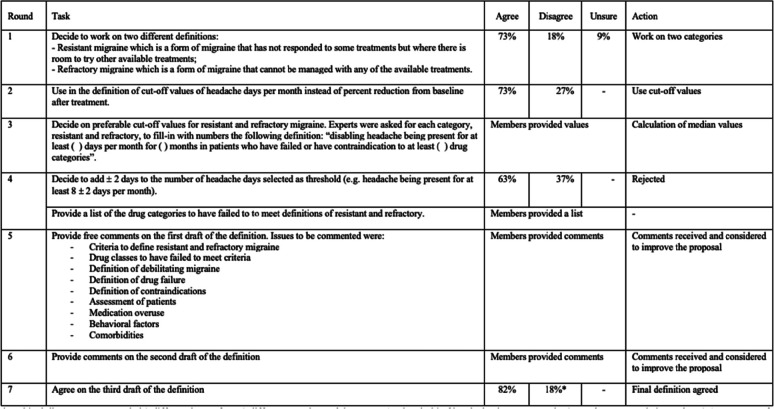
Rounds of the consensus process. After each round and before the next one, anonymized results and comments were shared. *Residual disagreement regarded 2 different issues from 2 different members of the group: 1. Threshold of headache days per month: 6 was the suggested alternative; 2 Assessment of patients: no need to be assessed by headache centers

## Results

Two categories of difficult to treat migraine were recognized, *resistant migraine* and *refractory migraine*. Criteria to define resistant and refractory migraine are reported in Table 3.

**Table 3:**
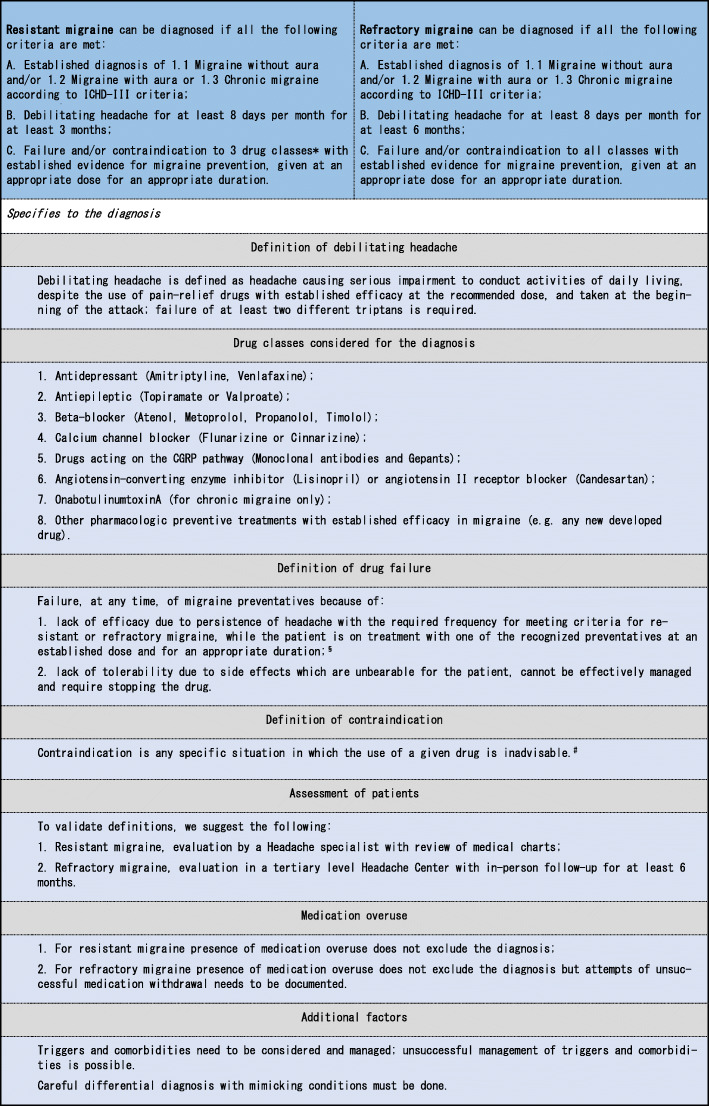
Proposed updated definition of resistant and refractory migraine

^a^Trials with at least 3 drug classes is encouraged if allowed by contraindications

^b^Established doses and duration as reported in Table 4

^c^Absolute, major and relative contraindications as reported in Table 5

Definitions are based on fulfilling ICHD III criteria for migraine with or without aura or for chronic migraine [7] and having at least 8 debilitating headache days per month plus the failure of previous preventive treatments.

*Resistant migraine* is defined by having failed at least 3 classes of migraine preventatives and suffer from at least 8 debilitating headache days per month for at least 3 consecutive months without improvement; definition can be based on review of medical charts.

*Refractory migraine* is defined by having failed all of the available preventatives and suffer from at least 8 debilitating headache days per month for at least 6 consecutive months. For refractory migraine, chart review alone is not sufficient and a minimum period of observation of 6 months is required together with completed diaries.

The drug classes considered for meeting the definitions include antidepressants, antiepileptics, beta-blockers, calcium channel blockers, drugs acting on the calcitonin-gene related peptide (CGRP) pathway, angiotensin-converting enzyme inhibitors or angiotensin II receptor blockers, onabotulinumtoxinA, as well as any new developed drug with established efficacy in migraine prevention. Drug failure may include lack of efficacy or lack of tolerability. For lack of efficacy, appropriate dosing and duration of the considered preventatives is reported in Table 4.

**Table 4:**
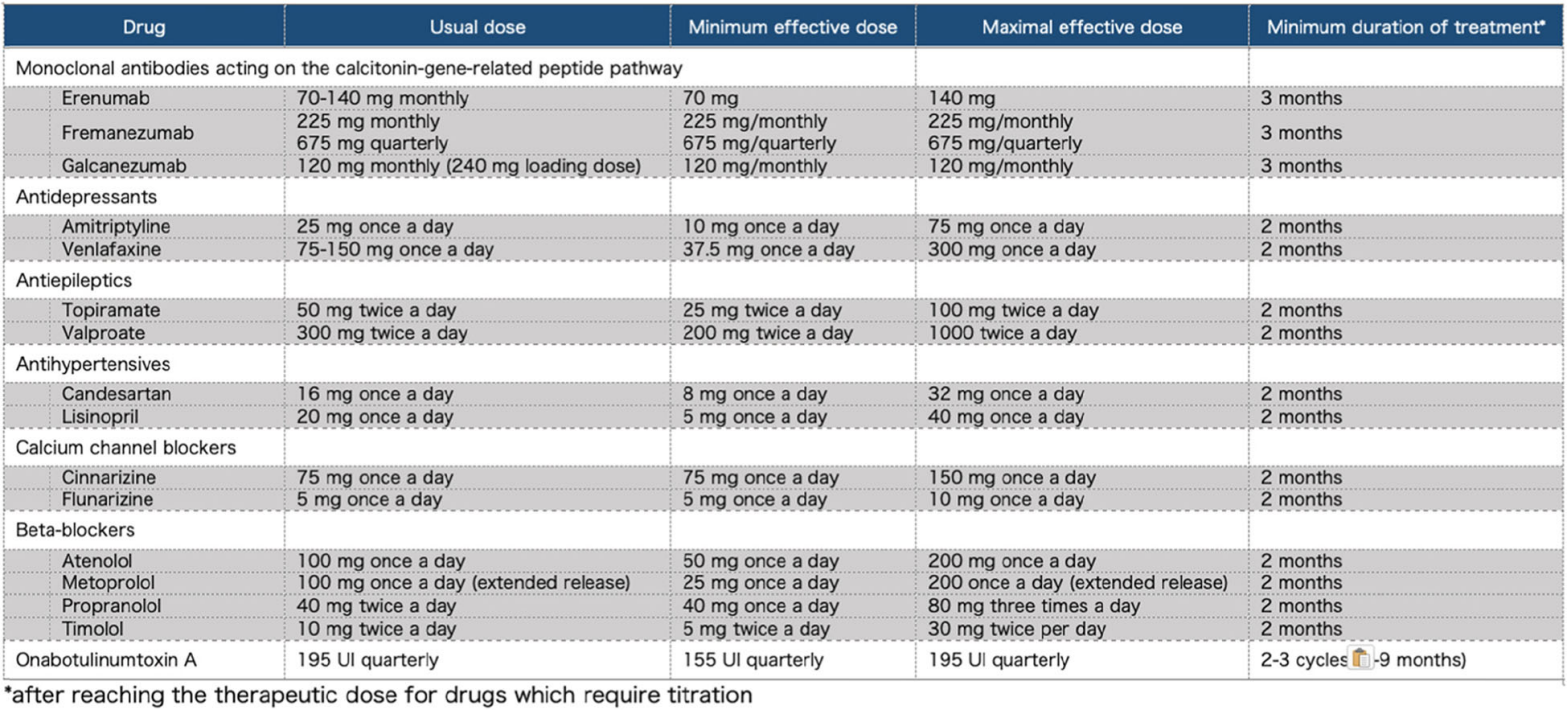
Suggested doses and duration for assessment of lack of efficacy

A patient cannot be considered to try some drugs because of possible contraindications. Absolute, major and relative contraindications to drugs for migraine prevention are reported in Table 5.

**Table 5:**
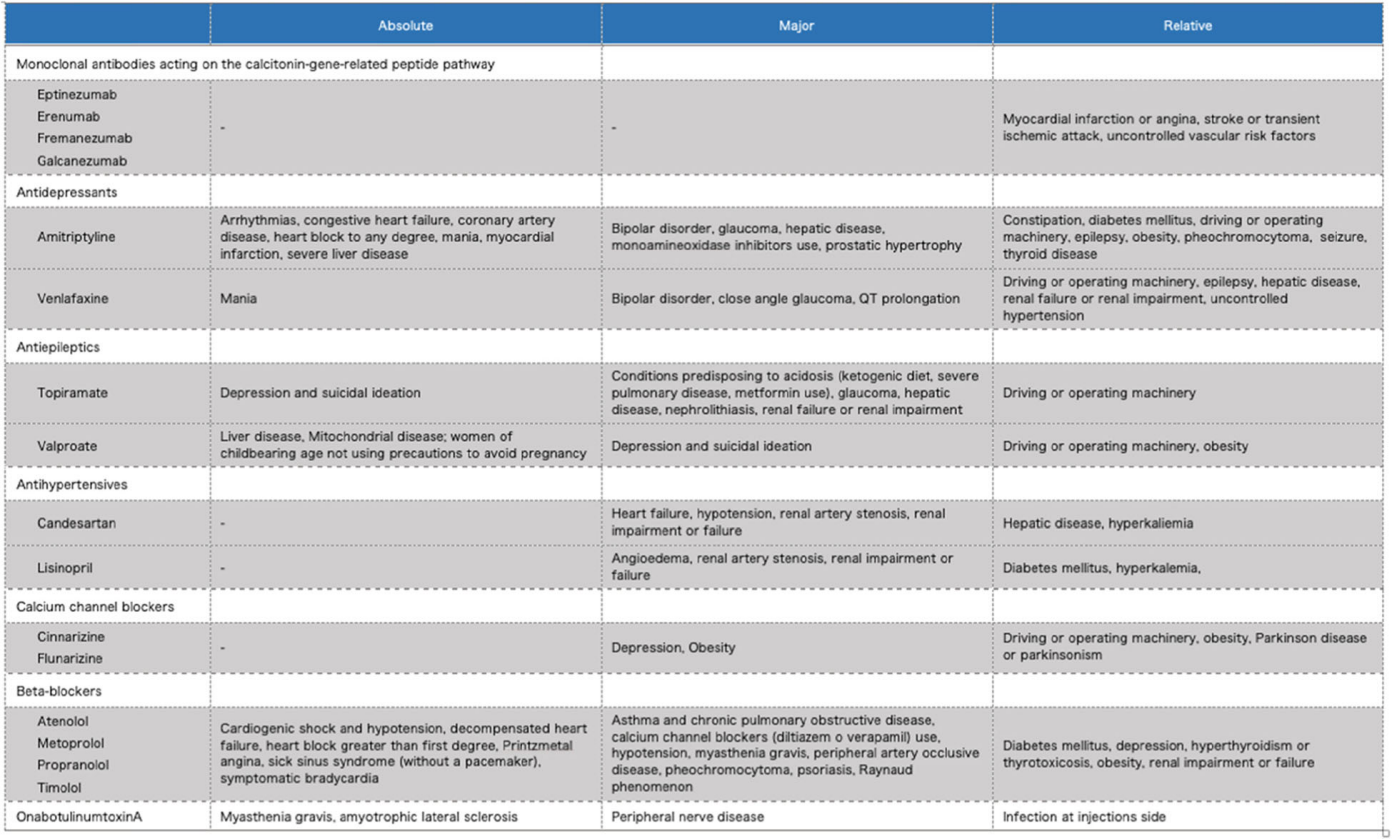
Absolute, major and relative contraindications to drugs for migraine prevention

The presence of medication overuse is compatible with the proposed definition of resistant and refractory migraine; however, in patients with refractory migraine documentation of failed attempts of withdrawal of medications is needed.

Both definitions of resistant and refractory migraine require the presence of at least 8 days of debilitating migraine. Debilitating headache is defined as headache causing serious impairment to conduct activities of daily living despite the use of pain-relief drugs with established efficacy at the recommended dose and taken early during the attack; failure of at least two different triptans is required.

To validate the definitions, it is also recommended that patients with resistant migraine are evaluated by headache specialists and that patients with refractory migraine are evaluated in tertiary Headache Centers.

Triggers and comorbidities which may contribute to resistant or refractory migraine need to be identified and managed before assigning patients to those categories. Careful differential diagnosis with mimicking conditions must be done as suggested in Table 6.

**Table 6:**
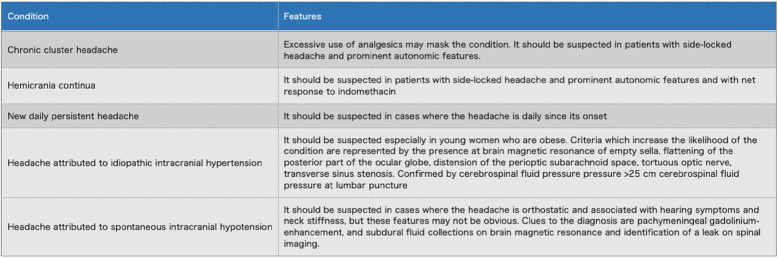
Conditions to be excluded before diagnosis of resistant or refractory migraine

## Discussion

### Resistant and refractory

It is necessary to distinguish between patients who easily respond to treatment and those who do not. The latter requires more attention and more persistence in the treatment. Moreover, patients who do not easily respond to treatment are at higher risk of developing chronic headache and therefore should be identified early and treated accordingly [10].

One of the major novelties of the updated EHF Consensus definition is the recognition that difficult-to-treat patients may be labelled into two major categories, *resistant migraine* and *refractory migraine;* the difference between resistant and refractory migraine relies on the number of required previous treatment failures.

The two categories were identified in order to respond to different needs and to implement these needs in the clinical and research settings. In fact, the optimal definition of difficult to treat migraine depends on the context and on the consequences of assigning the label. If the consequence of labeling is referral to a headache center or treatment escalation, the definition threshold should be lower than if the consequence is to develop new interventional treatments. Accordingly, resistant migraine is defined as migraine which remains significantly debilitating despite some treatment attempts. In contrast, refractory migraine is defined as migraine which remains significantly debilitating after maximal or nearly maximal number of treatment attempts. It is important to note that resistance and refractoriness may just represent more or less treatable versions of migraine attacks, rather than a separate migraine entity. The same patient might be identified as refractory at one time, but treatment responsive at another. Therefore, it may be of crucial importance to evaluate acute and prophylactic treatment response, baseline headache severity, partial response versus an all-or-none response, and the possibility of any variability in the treatment response over time [11].

The categorization of difficult-to-treat patients might be helpful in the recognition of patients in need of more expensive treatments. Additionally, the definition could help patients to gain access to reimbursement policies, work-related disability benefits, and social security benefits.

Anyhow, we duly recognize that patients who also do not meet criteria for resistant migraine (e.g. 6 debilitating migraine days per month) may need access to dedicated headache care or treatment escalation.

### Cut-offs values

A further main novelty of the present definition is the use of cut-off values rather than percent reductions. The use of cut-off values is more in line with the ICHD III criteria while the use of percentage reductions is more in line with clinical trial criteria. Clinical trials for preventive treatment define responders as those achieving at least 50% reduction in monthly migraine/headache days with treatment as compared to baseline [12]. For chronic migraine a 30% reduction is accepted [13]. However, with the use of percent reduction, patients may be considered responders but still have a relevant number of debilitating days with headache. To overcome this problem the EHF Expert Consensus Group opted to use 8 days per month as cut-off values. Eight days per month were chosen to consider evidence indicating that moderate disability starts after 4 migraine days per month [14–16]. Thus, the selected cut-off values allow us to consider both, episodic and chronic migraine as resistant or refractory. It is important to note that with the use of cut-off values, patients who have significant response to treatment (e.g a patient with 30 headache days per month and with > 50% reduction in headache days with treatment) may be labelled as resistant or refractory. With advancements in migraine treatment, goals of successful treatment may become more and more ambitious and cut-off values may need to be further reduced.

### Debilitating headache

The criteria proposed by the EHF Expert Consensus Group rely on the presence of a given number of days of debilitating headache attacks. The presence of a serious impairment in activities is a key feature of the proposed definitions. Debilitating headache was defined as headache causing serious impairment to the conduct of activities in daily living despite the use of pain-relief drugs with established efficacy at the recommended dose [17]. In order to assess the efficacy of acute headache medications, adequate timing of drug administration, dosage and formulations used, should be considered.

We opted for headache days rather than migraine days because overt migrainous features may be masked in complex patients especially in patients who overuse acute headache medication. We also voluntarily decided to use the term debilitating rather than disabling. The term disabling is more related to physical impairment caused by neurological disease such as stroke, multiple sclerosis, amyotrophic lateral sclerosis and others which cause permanent impairment in function of patients due to physical disabilities. As the impairment related to migraine attacks is temporary if attacks are not frequent or not always severe and due to cognitive impairment and head pain but not to physical disability, a different terminology may be more appropriate. However, we are aware that patients who suffer from migraine live with anticipatory anxiety as they do not know when the next attack may occur. This fear conditions their life and that of their families, social activities and work responsibilities [18].

Ascertainment of debilitating attacks shall be done with clinical interview or with the use of validated scales in order to evaluate if headaches significantly interfere with a patient’s ability to work, attend school, or participate in family or social activities. The EHF Expert Consensus Group did not choose to use cut scores at common validated instruments to measure function or disability such as the MIgraine Disability ASsessment (MIDAS) tool [19], the Headache Impact Test (HIT-6) scale [20], or the Headache-Attributed Lost Time (HALT) Indices [21]. We considered the fact that there is not one single Patient-Reported Outcome scale (PROs) including the magnitude of the impairment that a person with a migraine attack or frequent migraine suffers. This is, in a large part, due to the impact of migraine on cognition. When the brain can’t function, there is a wide impact on many different tasks which need a wide range of instruments to capture the complexity of the disability. Indeed, the recent randomized controlled trials on migraine prevention used several tools to capture the impact of both, the disease and the treatment [22, 23]. Additionally, the choice of any cut-off score might be controversial as there are no data regarding which threshold of score change in these instruments should separate treatment failure from treatment success. Moreover, the disability instruments may not be translated or validated in certain countries. However, when available, using PROs not only helps to translate burden of disease but also tracks changes over time.

In the proposed definition, the efficacy of analgesics is also considered. In fact, resistance or refractoriness to acute or preventive treatment, are not to necessarily related. One patient may have a poor response to preventatives but a good response to analgesics. Failure of at least two triptans is required because there is evidence that switching from a triptan that is ineffective to a second one can result in positive treatment [24]. If the analgesics are able to control adequately the pain allowing patients to function normally, patients with migraine will not qualify to be categorized as resistant or refractory. It is worth mentioning that according to the proposed definitions of resistant and refractory migraine, patients who overuse symptomatic drugs and who have pain relief with them cannot be labelled as resistant or refractory.

### Drug failure

Establishing drug failure or contraindication is pivotal to identifying resistant or refractory migraine. This point should carefully be addressed in order to avoid labelling as resistant or refractory patients who are only pseudo-resistant or pseudo-refractory.

One of the main criteria to define resistance and refractoriness to treatment is the number of failed classes of medications. The specific classes were picked because they have shown clinical efficacy in randomized-controlled trials and are supported by evidence-based guidelines for migraine prevention [13, 22, 25, 26]. The Consensus group agreed to select failure of drug classes, rather than single medications, since a patient who fails a drug has a very low probability of responding to another drug within the same class based on expert opinion. However, it is important to note that different agents within a class may work by different mechanisms [2, 27] and thus we support the use of drugs in the same class, but with a different mechanism of action (e.g. topiramate and valproate). We acknowledge that some agents may be not available or accessible across all countries and that others require technical expertise (e.g. OnabotulinumtoxinA), which may limit the generalizability of the proposed definitions. National adaptations of the EHF Consensus on the definition of resistant and refractory migraine can be developed for use at the country level.

The proposed definitions of resistant and refractory migraine consider the appropriateness of doing trials with preventive treatments. Identification of factors that contribute to pseudo-resistance is important in preventing costly and potentially risky diagnostic evaluations and in avoiding inappropriate intensification of treatment. If therapy was inadequate (low dose or incorrect duration) the failure is to be reassessed. Suggested doses and durations for optimal treatment are specified in Table 4. Minimal duration of drug trials was set at 2 months (after reaching the therapeutic dose) for oral drugs in line with previous definition of refractory migraine. For monoclonal antibodies (mAbs) acting on the CGRP pathway a minimum of 3 months is required and for OnabotulinumtoxinA a minimum of 6 months, in line with available guidelines and evidence from real-life studies [13, 22, 25, 28]. Longer trials would be preferable for some drugs, but extending minimal time frame is not feasible in every case, especially in those patients who do not have any kind of improvement with the selected preventatives. On the other hand, we may consider to extend the preventive treatment if there was some improvement during the first period of treatment. Undoubtedly, decisions will have to be individualized. The EHF Expert Consensus Group decided not to pose a time frame for previous trials of preventatives (e.g. failure within the 10 previous years) as any choice would be arbitrary.

It is important to note that lack of adherence may contribute to lack of success of medical treatment. Lack of adherence is mostly due to side effects. Frequently, side effects are too bothersome for patients and lead to drug discontinuation. EHF criteria for resistant and refractory migraine include lack of tolerability among the accepted reasons of drug failure. Anyway, we want to point out that side effects should always be addressed and if possible adequately managed to promote drug adherence. A trusting physician–patient relationship, setting reasonable goals and expectations, and educating patients are key for a successful patient management. Nocebo effect may lead patients to discontinue medications because of the fear of a potential side effect [29, 30].

Some patients have medical contraindications to specific preventive treatments (Table 5). Contraindicated drugs are considered in the criteria for resistant and refractory migraine. It is important to differentiate absolute contraindications that pose a risk for the patient if the drug is used, from a relative contraindication, which does not prohibit the use of the drug but may require stricter monitoring. One limit of the current definition is that a patient can be defined as resistant while not having tried any preventive medication because of hypothetical contraindications to at least three classes.

In the current EHF definitions of resistant and refractory migraine, we did not include in the criteria the possibility to combine different preventatives to achieve a response to treatment in the criteria. Combinations of suggested preventive treatments are recommended especially when one preventive treatment decreased the attack frequency but did not control the situation satisfactorily, upon the physician’s decision [31]. Combination therapy has the potential advantage to target different aspects of the pain dysfunction, with potentially better results due to the synergistic effects of different treatments. However, there is insufficient evidence to state definitively if combination therapy is clearly superior to single therapy [32–34].

### Medication overuse

It is well known that a variable proportion of patients overusing acute headache medications may actually benefit from withdrawal [35]. However, the ideal treatment strategy for medication overuse is a matter of ongoing debate. Withdrawal cannot be achieved in every patient and relapse or continued overuse has been reported in many patients [36]. For those reasons we opted to make the presence of medication overuse compatible with the proposed definition of resistant and refractory migraine. Up to know it is unclear if withdrawal of acute medication may revert an apparent refractory headache into a tractable one. Having a well-defined definition of refractory and resistant headache will represent the basis for research studies to establish this.

### Triggers and comorbidities

In some cases, difficult-to-treat migraine is associated with uncontrolled trigger factors (e.g. excessive use of caffeine) and comorbidities. The role that those factors have in drug resistance is not entirely clear. On one hand, they may contribute to resistance or refractoriness to prescribed pharmacological treatment but on the other hand if medications are effective, triggers may not be able to induce migraine attacks. Triggers and comorbidities should be addressed and managed before labelling patients as resistant or refractory, even if definite removal is not always possible.

Trigger factors that can exaggerate migraine include alcohol consumption, caffeine overuse, diet, smoking and vasodilating antihypertensives. Further triggers are represented by emotional stress [37], alterations in the sleep cycle [38–40], and hormonal factors [41–43]. Management of those factors is a staple of good clinical practice. Additionally, patients suffering from migraine frequently have comorbid disorders [44–48]. The identification and management of all clinically significant comorbidities is essential before declaring a treatment failure in migraine patients. Psychiatric comorbidities and obesity may be particularly relevant in the setting of resistance or refractoriness to treatments [49, 50].

### Differential diagnosis

A possible reason for treatment failure in patients with headache is that the diagnosis is incomplete or incorrect. When addressing patients with migraine who do not respond to treatment it is always important to reconsider the diagnosis and to rule out other primary or secondary headaches, which may entirely account for the clinical picture or which may coexist with migraine and contribute to the lack of response to treatments. Investigations should be done where appropriate to exclude secondary headaches [51]. A list of conditions mimicking resistant or refractory migraine is reported in Table 6.

Particular attention should be paid to idiopathic intracranial hypertension without papilledema (IIIWP). This condition can be entirely responsible of difficult-to-treat headache or can contribute to drug resistance or refractoriness in patients who have concomitant migraine. Criteria which increase the likelihood of IIIWP are represented by the presence, at brain magnetic resonance, of empty sella, flattening of the posterior part of the ocular globe, distension of the perioptic subarachnoid space, tortuous optic nerve, transverse sinus stenosis [52]. If this IIIWP is suspected, a lumbar puncture with measurement of cerebrospinal fluid pressure should be performed and patients treated according to available guidelines [52].

### Other limitations to the definition

Migraine is a cyclic disorder which tends to fluctuate in frequency, severity and disability during life. Specifically, this has been investigated over the course of a year and for example, patients with chronic migraine can fluctuate into an episodic migraine phase [53]. This is why these new proposed criteria for the definition of resistant and refractory migraine have considered following also the International Classification for Headache Disorders, three consecutive months as a time frame to define at that time point in life, the presence of a resistant or refractory migraine. However, a diagnosis is not static in headache, and can change over time. If this should happen, it can always be revised.

It is also important to note that the present definition did not consider failure to devices or non-pharmacological therapies. A number of studies have shown that vagal nerve stimulation, biofeedback, relaxation, and cognitive behavior therapy are as efficacious for migraine as placebo, but still better than no improvement [54, 55]. However, behavioral treatments are less accessible, more professional-dependent and less standardized than pharmacological treatments, and in certain countries, not reimbursed by the Healthcare systems, than pharmacological treatments.

Moreover, physician-patient relationship, lifestyle changes, and other qualitative variables can also influence the response to treatment.

### Assessment of patients

Migraine in general can be managed in the setting of primary care [56] or general neurology [57]. However, such settings cannot provide the necessary expertise to treat resistant and refractory patients. Patients with resistant migraine should be managed in special interest headache care settings (e.g. general practitioners with special interest in headache or neurologists, or first/second level headache clinics) and patients with refractory migraine must be managed in tertiary level headache clinics [58, 59]. In particular, refractory migraine patients must be offered the possibility of access to a multidisciplinary team.

Management by non-headache specialists often leads to perform-and-repeat exams which are not needed, thus raising the cost of migraine care [60, 61]. Additionally, incidental findings to unnecessary exams may lead to over-treatment of some conditions (e.g patent foramen closure) or additional unnecessary examinations (e.g. thrombophilia screening in unselected patients with white matter abnormalities).

Establishing previous treatment failures ideally should be done by chart review. It is not reasonable to test again preventive medications used in the past with no efficacy or with intolerable side effects provided that information on duration, dose and adverse events is sufficient and reliable. Unfortunately, not all patients provide charts and headache diaries. In those cases, careful collection of information from patient and relatives should be looked for with the help of the general practitioner, if available.

For patients with refractory migraine who were never followed by a headache specialist, a minimum follow-up of 6 months is suggested, to obtain reliable diaries and allows the possibility to.

further improve headache management, as the diagnosis of refractory migraine is a very serious matter, which should be carefully established.

### Implications to be defined as resistant or refractory migraine patient

The diagnosis of resistant or refractory migraine may contribute to stigmatizing the patient and therefore may have profound psychological implications [62]. This is especially true because the concept seems static and unresolvable. Education and effort will have to be put in with both treating physicians and patients to help them to understand the possible fluctuations of the disease.

There could also be legal and healthcare implications. It may be possible that in certain countries only those patients with a resistant or refractory migraine may have access to newer treatments such as monoclonal Antibodies (mAbs) acting on the Calcitonin-Gene-Related-Peptide (CGRP) pathway; or in other countries, it might allow for temporary or permanent social welfare [14, 63]. Even if revisable, it does support a difficult-to-treat disorder and should facilitate access to more resources for its management.

### Future perspectives

The introduction of the concept of resistant and refractory migraine will have clinical and political implications. We attempt to provide operational criteria of what intuitively has been used already. Introduction of expensive migraine preventatives such as OnabotulinumtoxinA and mAbs acting on the CGRP pathway and the expectation of further preventatives raised the question of cost effectiveness of these treatments. In many European Countries use of these treatments is restricted to the difficult-to-treat subpopulations of migraine patients. These subpopulations were defined based on frequency (e.g. in Spain more than 8 headache days per month) or non-response (e.g. in Germany more than 5 preventatives for episodic migraine and 6 preventatives including OnabotulinumtoxinA for chronic migraine). This manuscript aims to provide solid expert-opinion based criteria for these subpopulations. It will be important to have adequate field-testing of the proposed updated EHF definition of resistant and refractory migraine in order to identify possible deficiencies and to make further improvements. Field-testing should be performed across different countries and different clinical settings. Adaptations at the country level may be needed to make the definition more usable.

Accepted and reliable criteria represents the basis of further research. At present, the exact pathophysiology of refractory migraine is unknown. It is important to reliably identify factors which may lead to refractoriness. We do not know whether genetic predisposition plays a role in resistance to treatment, whether resistance is accompanied over time by changes in the brain structure and function as assessed by neuroimaging, or which mechanisms play a pivotal role (e.g. central sensitization, peripheral sensitization).

There are several migraine preventive treatments in development; the proposed criteria will allow inclusion of these treatments when there is a good evidence basis for their use. Criteria will need to be changed when new treatments and significant advancements in the migraine field will happen.

## Conclusion

Crafting an operational definition of so called “difficult-to-treat migraine” is challenging. However, we all need to be aware that these patients exist and that a good definition represents a major need for clinical practice, for legal issues, and for research purposes. The proposed updated EHF definition identifies two subsets of difficult-to-treat migraine, resistant and refractory migraine, and considers both frequency and disability from single and frequent attacks. Although, in the previous literature formal and operational definitions for refractory or intractable headache were proposed, none was universally accepted. We hope, that the updated EHF definition will be able to solve the conflicts that have limited the use of definitions which have been put forward in the past. Only with a widely accepted definition, progresses in difficult-to-treat migraine can be achieved, underlying mechanisms can be identified, epidemiology can be characterized, and evidence-based treatments can be developed. Furthermore, the aim of this new definition is to increase the understanding of the impact of migraine as a disease with all of its social, legal and healthcare implications. It is the hope of the EHF Expert Consensus Group that the proposed criteria will stimulate further clinical, scientific and social attention to patients who suffer from migraine which is difficult-to-treat.

## Data Availability

Not applicable.

## Abbreviations

CGRP: Calcitonin-gene related peptide
EHF: European Headache Federation
EHMA: European Migraine & Headache Alliance
HALT: Headache-Attributed Lost Time
HIT-6: Headache Impact Test
ICHD: International Classification of Headache Disorders
IIIWP: Idiopathic intracranial hypertension without papilledema
mAbs: Monoclonal antibodies
MIDAS: MIgraine Disability ASsessment
PROs: Patient-Reported Outcome scale
